# Harm reduction and recovery services support (HRRSS) to mitigate the opioid overdose epidemic in a rural community

**DOI:** 10.1186/s13011-023-00532-3

**Published:** 2023-04-19

**Authors:** Moonseong Heo, Taylor Beachler, Laksika B. Sivaraj, Hui-Lin Tsai, Ashlyn Chea, Avish Patel, Alain H. Litwin, T. Aaron Zeller

**Affiliations:** 1 Department of Public Health Sciences, Clemson University, Clemson, SC 29634 USA; 2 Prisma Health Addiction Medicine Center, Greenville, SC 29605 USA; 3 University of South Carolina School of Medicine—Greenville, Greenville, SC 29605 USA; 4 University of South Carolina School of Medicine—Columbia, Columbia, SC 29209 USA; 5 Department of Medicine, Prisma Health, Greenville, SC 29605 USA; 6 Clemson University School of Health Research, Greenville, SC 29605 USA; 7 Seneca Family Medicine Residency Program, Prisma Health Oconee Memorial Hospital, 139 Lila Doyle Drive, Seneca, SC 29672 USA

**Keywords:** Social capital, Rural, Overdose, Opioid use disorder, Recovery, Harm reduction

## Abstract

**Background:**

Rural areas in the United States (US) are ravaged by the opioid overdose epidemic. Oconee County, an entirely rural county in northwest South Carolina, is likewise severely affected. Lack of harm reduction and recovery resources (e.g., social capital) that could mitigate the worst outcomes may be exacerbating the problem. We aimed to identify demographic and other factors associated with support for harm reduction and recovery services in the community.

**Methods:**

The Oconee County Opioid Response Taskforce conducted a 46-item survey targeting a general population between May and June in 2022, which was mainly distributed through social media networks. The survey included demographic factors and assessed attitudes and beliefs toward individuals with opioid use disorder (OUD) and medications for OUD, and support for harm reduction and recovery services, such as syringe services programs and safe consumption sites. We developed a Harm Reduction and Recovery Support Score (HRRSS), a composite score of nine items ranging from 0 to 9 to measure level of support for placement of naloxone in public places and harm reduction and recovery service sites. Primary statistical analysis using general linear regression models tested significance of differences in HRRSS between groups defined by item responses adjusting for demographic factors.

**Results:**

There were 338 survey responses: 67.5% were females, 52.1% were 55 years old or older, 87.3% were Whites, 83.1% were non-Hispanic, 53.0% were employed, and 53.8% had household income greater than US$50,000. The overall HRRSS was relatively low at a mean of 4.1 (SD = 2.3). Younger and employed respondents had significantly greater HRRSS. Among nine significant factors associated with HRRSS after adjusting for demographic factors, agreement that OUD is a disease had the greatest adjusted mean difference in HRSSS (adjusted diff = 1.22, 95% CI=(0.64, 1.80), p < 0.001), followed by effectiveness of medications for OUD (adjusted diff = 1.11, 95%CI=(0.50, 1.71), p < 0.001).

**Conclusions:**

Low HRRSS indicates low levels of acceptance of harm reduction potentially impacting both intangible and tangible social capital as it relates to mitigation of the opioid overdose epidemic. Increasing community awareness of the disease model of OUD and the effectiveness of medications for OUD, especially among older and unemployed populations, could be a step toward improving community uptake of the harm reduction and recovery service resources critical to individual recovery efforts.

## Introduction

Rural areas in the United States (US) are ravaged by the opioid epidemic [1, 2]. Residents of rural Appalachia are 43% more likely than the rest of the US to die from a drug overdose [3]. The rural opioid overdose epidemic has not spared Oconee County, which is located in the northwestern corner of South Carolina, and is designated as an entirely rural Appalachian County by the US Census Bureau [4]. The overdose death rate in Oconee County in 2020 was 18.9 deaths per 100,000, [5] and the South Carolina state overdose death rate was 34.9 per 100,000, which was ranked as the 17th highest among 50 US states and Washington DC [6]. While the underlying reasons for the high risk of opioid overdose mortality in Oconee County are unknown, excess availability of substances, a risk factor for misuse, [7] was noted by a US Drug Enforcement Administration report, which indicated that a pharmacy in Oconee County receives the 4th highest number of prescription opioids in the state [8]. Additionally, stigma towards persons with opioid use disorder (OUD) manifests in low rates of willingness to help someone experiencing addiction or overdose [9] and fits the pattern of low social capital seen in rural communities [10–12].

A single definition of social capital is not well established [13–16]. It has been defined as the ability of people to work together for common purposes in groups and organizations, [17–19] and as a capability that arises from the prevalence of trust in a society or community, with trust being the expectation of regular, honest, and cooperative behavior from other members of a community [20, 21]. Recent literature has continued to emphasize the trust and collaboration that are inherent to a solid definition of social capital [22]. In addition to these less tangible aspects of social capital, a tangible definition of social capital has been undertaken, and includes such metrics as the number of establishments in religious organizations, civic and social associations, business associations, political organizations, non-profit organizations with a local/regional mission, athletic clubs, and the like [12, 23]. So while social capital has been rightly described as a collective manifestation of the behaviors, attitudes, and values of individual members of a community, [12] it is also more than these attributes. These attributes should ultimately manifest themselves in support for and participation in community-level institutions. When these different perspectives on social capital are combined, a holistic definition emerges: social capital is a capability arising from both: (1) the intangible attributes within a group or community – including trust and a willingness to collaborate to achieve a common goal; and (2) the presence of tangible social assets – institutions capable of improving the life of the community.

Large swaths of the country are negatively affected by low social capital, including many rural areas, the South, and Appalachia [12]. Exploring the reasons for this phenomenon falls outside the scope of this study, but several references are included for interested readers [24–26]. A community with low social capital will find itself at cross-purposes as it attempts to solve problems like the opioid overdose epidemic [27]. While the perception that addiction is a failure of morals or of willpower has long plagued America’s approach to the opioid epidemic, [28] increased social capital in communities has been linked to improved protection from drug overdose [29]. Still, ideologies and attitudes that penalize cooperation and solidarity (e.g., low levels of intangible social capital) result in a lack of community resources that might help neighbors experiencing poverty, addiction, mental health conditions, homelessness, and other societal ills (e.g., lack of tangible social capital). Low social capital creates a vicious cycle, or Fukuyama’s ‘distrust tax’, [20] as those most in need of the community’s help end up stigmatized, marginalized, and ignored, the problem becomes larger, and the community feels even less safe.

Studies have shown that inadequate social capital at a societal level is associated with increased prevalence of OUD [11, 29, 30]. Since low social capital fuels the OUD epidemic and its worst outcomes, improving both the intangible and tangible aspects of social capital that directly address the opioid overdose epidemic will attack it at one of its roots and break the vicious cycle keeping OUD so prevalent in rural communities. Harm reduction and recovery support services fall into the category of tangible social capital as non-profit organizations with the mission of improving the well-being and health of the community.

To combat the local effects of the opioid epidemic, Oconee County Opioid Response Taskforce (OCORT) was formed in 2019 by multiple community stakeholders, including the Prisma Health Addiction Medicine Center. OCORT members include representatives from local law enforcement, emergency medical services, primary care practices, county administration, the school district, and the departments of health and social services, treatment and recovery organizations, and concerned members of the community. To assess community needs and make a strategic plan to address the most pressing opioid epidemic issues, OCORT conducted a comprehensive community needs assessment in 2019 by disseminating a 24-item survey to county residents to measure stigma, knowledge, and opportunities for interventions and educational initiatives pertinent to OUD [9]. This survey was updated, revised and expanded in 2022 with newly added demographic items and additional recovery and harm reduction services items to assess support levels for organizations capable of mitigating the opioid overdose epidemic.

In this study, using the OCORT 2022 survey responses, we aimed to identify demographic factors and knowledge, beliefs, and attitudes toward harm reduction and recovery support that, if improved, might increase support for community resources to mitigate the opioid overdose epidemic.

## Methods

### Setting and design

Oconee County has a population of 78,314 with Whites composing 82.3% of the population according to the 2020 census. With respect to tangible social capital, the county has one hospital, Prisma Health Oconee Memorial Hospital, two community improvement organizations, three family support and assistance organizations, organizations for poverty and hunger, and 34 churches [31]. Although some of these organizations provide support for individuals with OUD, the county is currently lacking in resources intended to reduce substance use disorders (SUD) and mitigate the opioid overdose epidemic.

The 2022 OCORT Community Attitudes Survey had a total of 46 items, inclusive of demographic factors, and elicited beliefs and attitudes toward individuals with OUD and medications for OUD (MOUD), in addition to SUD knowledge, and support for harm-reduction services. The survey was disseminated via online methods using the REDCap platform and solicitation of paper surveys which were then entered into the REDCap portal by research assistants. The survey was disseminated between May and June 2022 to the general population of Oconee County. To this end, it was distributed through social media marketing on Facebook pages run by OCORT members, via Prisma Health emailing lists targeted to Oconee County zip codes, and through direct solicitation of participants by research assistants in 4 primary care offices throughout the county on a set schedule throughout the month. The survey received a Prisma Health Institutional Review Board exemption.

### Demographic factors

The survey items included the following demographic factors, and we dichotomized multiple responses of each item in the following manner for between-group comparisons: Age (Younger than 55 vs. 55 or older), Sex (Male vs. Female), Race (Whites vs. African-American/Asian or Pacific Islander/Native American/Two or More/Other), Ethnicity (Hispanic vs. non-Hispanic), Marital Status (Married vs. Divorced/Single/Widowed/Separated), Employment (Employed full/part time vs. Seeking/Retired), Household Income (< US$50,000 vs. ≥US$50,000). Item responses with “prefer not to answer” were treated as missing.

### Harm reduction and recovery support score outcome

We developed an outcome to measure a level of support for OUD recovery and for building harm reduction service resources. This composite score included 2 items addressing recovery and 7 items addressing harm reduction services. The score was calculated as the number of “Agree” responses to these 9 items. This composite score is herein referred to as “Harm Reduction and Recovery Support Score” (HRRSS). The score ranged from 0 to 9 with the internal consistency estimated by a Cronbach alpha as 0.72 (95%CI = (0.66, 0.78)). The HRRSS items included:

1. Emergency naloxone boxes should be placed in public places for emergency response to overdose (placement of emergency naloxone).
2. I would support provision of MOUD in the incarcerated population (support for MOUD among the incarcerated).
3. I would support HIV and Hepatitis C screening in the county (support for HIV and HCV screening).
4. I would support condom distribution in the county (support for condom distribution).
5. I would support having a syringe service program (SSP) in my neighborhood (support for SSP located in neighborhood).
6. I would provide financial support for a syringe service program (financial support for SSP).
7. I would support the syringe service program by utilizing the services they provide (Support for utilization of SSP).
8. I would support the concept of syringe services in the community (Support for concept of SSP).
9. I would support safe consumption sites (SCSs), defined as health services where individuals can inject or consume substances in a hygienic environment under the supervision of trained staff, and have opportunities to engage in other health and social services (support for SCS).

We note that there were additional three items on support for MOUD: methadone, buprenorphine, and naltrexone. However, these items were not included in the above HRRSS since a factor analysis revealed that these items belonged to a factor different from that of the above harm reduction and recovery support items, possibly representing a different underlying construct.

### Survey item predictors

We considered 22 items related to OUD knowledge, attitudes, and beliefs related to OUD, individuals with OUD, and harm reduction as potential factors associated with the HRSSS outcomes. (Table 1). Two groups of each item were classified based on the response to each survey questionnaire item; the first and second groups consisted of respondents who agreed/true and those who disagreed/false, respectively. However, “positive” responses between agree and disagree depend on items and are not consistent across all items, and some item responses are neutral between them.

**Table 1:** Description of survey questionnaire items/factors potentially associated with recovery support

Item	Description of Questionnaire	Response
OUD as disease	An opioid use disorder is a real illness like diabetes and heart disease	Agree/ Disagree
Addiction to pain medications	Anyone can become addicted to pain medications	Agree/ Disagree
Can stop drug use	If a person is addicted to drugs, they can stop using if they really want to.	Agree/ Disagree
Possible to recover	It is possible to sustain recovery from opioid use disorder	Agree/ Disagree
Higher rate of SUD-affected newborn	There are higher rates of newborns affected by substance use disorders in our community than other communities in SC	Agree/ Disagree
Adequate resources for pregnant women with SUD	There are adequate resources for pregnant women with substance use disorders in our community	Agree/ Disagree
Abstinence based therapy	Abstinence based therapy is the only successful form of treatment for substance use disorders	Agree/ Disagree
Relapse of overdose	Individuals who receive rehabilitation or treatment will just overdose again	Agree/ Disagree
Willingness to live in neighborhood of OUD	I would willingly live in the same neighborhood as an individual with OUD	Agree/ Disagree
OUD only for low-income individuals	OUD only affects low-income individuals	Agree/ Disagree
Easily spotting OUD	I can easily spot an individual in my community with an opioid use disorder	Agree/ Disagree
Embarrassed to reveal individuals with OUD	I would be embarrassed to tell people that someone close to me has an opioid use disorder	Agree/ Disagree
OUD dangerous	Individuals with opioid use disorders are likely to be dangerous.	Agree/ Disagree
Same right to a job	An individual with an opioid use disorder should have the same right to a job right as anyone else	Agree/ Disagree
OUD part of supportive community	It is important for individuals with an opioid use disorder to be part of supportive community	Agree/ Disagree
Comprehensive prenatal care to pregnant women with SUD	Pregnant women with substance use disorders should have access to comprehensive prenatal care, including appropriate counseling and/or MOUD	Agree/ Disagree
Naloxone administration to a stranger	I would willingly administer naloxone to a stranger in an overdose situation	Agree/ Disagree
Every time naloxone administration to overdose	Naloxone should be administered to every individual experiencing overdose, every time	Agree/ Disagree
Country at risk for HIV or HCV outbreak	The county is at risk for an HIV and/or Hepatitis C outbreak.	Agree/ Disagree
Effectiveness of MOUD	Medication for opioid use disorder, specifically buprenorphine, methadone, and naltrexone, is an effective treatment for opioid use disorder	Agree/ Disagree
HCV screening offered	My doctor or nurse has offered to test me for Hepatitis C	True/ False
HIV screening offered	My doctor or nurse has offered to test me for HIV	True/ False

### Statistical analysis

Descriptive statistics included mean, standard deviation (SD), frequency and percentages of survey responses. Significance of difference in mean HRRSS between groups was tested by two-sample t-tests. To identify factors associated with HRRSS after adjusting for demographic factors, we tested significance of adjusted mean difference in HRRSS between groups of each candidate factor using a general linear regression model that included all demographic factors as adjusting covariates. We anticipated that at least N = 300 residents would respond to the survey to detect with > 80% statistical power a relatively small effect size of Cohen’s d (i.e., difference in means on a standard deviation (SD) unit scale) = 0.3 between groups categorized by survey responses. All statistical analysis was conducted using R software v.4.0.5 [32] with RStudio [33] and test results with a two-sided p-value < 0.05 were declared statistically significant.

## Results

### Participant demographic compositions

A total of 338 Oconee County residents responded to the survey. After excluding missing responses and those with “prefer not to answer” from the denominators, N = 228 (67.5%) were females, N = 176 (52.1%) were 55 years old or older, N = 295 (87.3%) were Whites, N = 281 (83.1%) were non-Hispanic, N = 207 (61.2%) were married, N = 179 (53.0%) were employed (89% (124/140) were retired among Seeking/Retired), N = 182 (53.8%) had household income of US$50,000 or more. (Table 2)

**Table 2:** Respondents’ characteristics and distributions of item responses

Demographic Characteristic	Subgroup	n (%)*
Age	12–54 years	161 (47.8%)
	55 years and above	176 (52.2%)
Gender	Female	228 (68.1%)
	Male	107 (31.9%)
Race	White	295 (88.6%)
	Other	38 (11.4%)
Ethnicity	Hispanic or Latino/a	9 (3.1%)
	Not Hispanic or Latino/a	281 (96.9%)
Marital status	Married	207 (62.4%)
	Other	125 (37.7%)
Employment status	Employed full time or part time	179 (56.1%)
	Other	140 (43.9%)
Household income	Less than $50,000	116 (38.9%)
	$50,000 or more	182 (61.1%)
	Response, n (%)*	
**Survey Items**	**Agree**	**Disagree**
OUD as disease	254 (76.5%)	78 (23.5%)
Addiction to pain medications	322 (96.1%)	13 (3.9%)
Can stop drug use	145 (44.8%)	179 (55.2%)
Possible to recover	309 (95.1%)	16 (4.9%)
Higher rate of SUD-affected newborn	169 (58.7%)	119 (41.3%)
Adequate resources for pregnant women with SUD	103 (35.0%)	191 (64.9%)
Abstinence based therapy only	93 (30.7%)	210 (69.3%)
Relapse of overdose	35 (11.2%)	278 (88.8%)
Willingness to live in neighborhood of OUD	153 (49.8%)	154 (50.2%)
OUD only for low-income individuals	24 (7.3%)	304 (92.7%)
Easily spotting OUD	76 (23.7%)	245 (76.3%)
Embarrassed to reveal individuals with OUD	88 (27.2%)	236 (72.8%)
OUD dangerous	133 (42.6%)	179 (57.4%)
Same right to a job	170 (54.8%)	140 (45.2%)
OUD part of supportive community	302 (94.1%)	19 (5.9%)
Comprehensive prenatal care to pregnant women with SUD	303 (95.3%)	15 (4.7%)
Naloxone administration to a stranger	226 (71.5%)	90 (28.5%)
Every time naloxone administration to overdose	188 (62.1%)	115 (37.9%)
Country at risk for HIV or HCV outbreak	154 (54.2%)	130 (45.8%)
Effectiveness of MOUD	195 (69.9%)	84 (30.1%)
	**True**	**False**
HCV screening offered	115 (36.9%)	197 (63.2%)
HIV screening offered	97 (31.2%)	214 (68.8%)

* The extents of missing responses and response with “prefer not to answer” depended on the items

### Distribution of predictor item responses

A vast majority of participants agreed that anyone can become addicted to pain medications (96.1%), that it is possible to sustain recovery from OUD (95.1%), that it is important for individuals with an opioid use disorder to be part of supportive community (94.1%), and that pregnant women with substance use disorders should have access to comprehensive prenatal care, including appropriate counseling and/or MOUD (95.3%). In contrast, a distinct minority of participants agreed that OUD only affects low-income individuals (7.3%), and that individuals who receive rehabilitation or treatment will just overdose again (11.2%). Approximately three quarters of the participants (76.5%) agreed that an opioid use disorder is a real illness like diabetes and heart disease. Approximately one quarter of the respondents (23.7%) agreed that they can easily spot an individual in their community with an opioid use disorder. The rates of “agree/true” responses of all the other items ranged between 25% and 75%. (Table 2)

### Distribution of positive responses of HRRSS items

The “Agree” response rates for individual HRRSS recovery support items were: 58.3% for placement of emergency naloxone, and 26.2% for HRRSS support for MOUD among the incarcerated. The ‘agree’ response rates for harm reduction services support items were: 90.7% for support for HIV and HCV screening, 82.9% for support for condom distribution, 21.0% for support for SSP in neighborhood, 11.5% for financial support for SSP, 10.1% for support for utilization of SSP, 42.3% for support for concept of SSP, 45.1% for support for safe consumption sites. The overall mean (SD) of the HRRSS was 4.1 (2.3).

### Difference in recovery support scores between subgroups of demographic factors

Younger respondents (< 55 years) had significantly greater mean HRRSS than older respondents (≥ 55 years) (4.9 (2.1) vs. 3.4 (2.1), p < 0.001). Respondents with full or part time employment respondents had a significantly greater mean HRRSS than those with other employment statuses (4.6 (2.2) vs. 3.5 (2.1), p < 0.001). Neither gender, ethnicity, race, marital status, nor income level was significantly associated with HRRSS. (Table 3)

**Table 3:** Harm reduction and recovery support score (HRRSS) between groups categorized by demographic factors

Characteristic	Subgroup	Mean (SD)	p
Age	12–54 years	4.94 (2.14)	**< 0.001**
	55 years and above	3.35 (2.11)	
Gender	Female	4.23 (2.18)	0.070
	Male	3.76 (2.35)	
Race	White	4.01 (2.30)	0.060
	Other	4.74 (1.80)	
Ethnicity	Hispanic or Latino/a	4.56 (1.24)	0.600
	Not Hispanic or Latino/a	4.18 (2.21)	
Marital status	Married	3.98 (2.18)	0.300
	Other	4.26 (2.36)	
Employment status	Employed full time or part time	4.56 (2.20)	**< 0.001**
	Other	3.51 (2.14)	
Household income	Less than $50,000	4.35 (2.32)	0.400
	$50,000 or more	4.14 (2.16)	

### Factors associated with HRRSS

The strongest factor associated with the HRRSS was the understanding of OUD as disease, “OUD as disease”, which has the greatest adjusted mean difference of HRRSS (adjusted diff = 1.22, 95%CI = (0.64, 1.80), p < 0.001) after adjusting for demographic factors. (Table 4) The “Effectiveness of MOUD” factor had the second greatest adjusted mean HRRSS difference of 1.11 (95%CI = (0.50, 1.71), p < 0.001). Other factors significantly associated with greater adjusted HRRSS scores are displayed in Table 4 as follows: agree on “Willingness to live in neighborhood of OUD”, “Same right to a job”, “OUD part of supportive community”, and “Naloxone administration to a stranger”; and disagree on “Can stop drug use”, “Abstinence therapy only”, and “OUD dangerous”.

**Table 4:** Effect of beliefs, attitudes, and knowledge on the HRRSS

Item	Mean (SD)			Adjusted diff* (95% CI)	p
	**Agree/True**	**Disagree/False**	**p**		
OUD as disease	4.46 (2.25)	3.13 (1.92)	**< 0.001**	1.22 (0.64, 1.80)	**< 0.001**
Addiction to pain medications	4.16 (2.24)	3.62 (2.63)	0.400	0.7 (-0.98, 2.39)	0.412
Can stop drug use	3.80 (2.05)	4.51 (2.35)	**0.005**	-0.58 (-1.10, -0.05)	**0.032**
Possible to recover	4.21 (2.24)	4.00 (2.19)	0.700	0.66 (-0.63, 1.95)	0.318
Higher rate of SUD-affected newborn	4.66 (2.26)	3.93 (2.07)	**0.006**	0.52 (-0.05, 1.09)	0.077
Adequate resources for pregnant women with SUD	4.03 (2.21)	4.54 (2.18)	0.058	-0.08 (-0.67, 0.51)	0.783
Abstinence based therapy only	3.63 (2.06)	4.67 (2.14)	**< 0.001**	-0.72 (-1.31, -0.12)	**0.019**
Relapse of overdose	4.17 (2.01)	4.36 (2.18)	0.600	0.06 (-0.77, 0.89)	0.891
Willingness to live in neighborhood of OUD	4.81 (2.32)	3.83 (1.91)	**< 0.001**	0.92 (0.40, 1.45)	**0.001**
OUD only for low-income individuals	4.25 (1.67)	4.22 (2.23)	0.943	0.18 (-0.75, 1.11)	0.707
Easily spotting OUD	4.26 (1.97)	4.26 (2.27)	0.982	-0.19 (-0.82, 0.45)	0.562
Embarrassed to reveal individuals with OUD	4.00 (1.88)	4.33 (2.28)	0.200	-0.39 (-0.97, 0.20)	0.196
OUD dangerous	3.62 (1.96)	4.78 (2.26)	**< 0.001**	-0.9 (-1.43, -0.37)	**0.001**
Same right to a job	4.79 (2.29)	3.61 (1.90)	**< 0.001**	1.05 (0.52, 1.58)	**< 0.001**
OUD part of supportive community	4.33 (2.20)	3.00 (1.63)	**0.010**	0.92 (-0.16, 1.99)	0.096
Comprehensive prenatal care to pregnant women with SUD	4.36 (2.15)	3.00 (2.14)	**0.017**	0.74 (-0.39, 1.88)	0.199
Naloxone administration to a stranger	4.55 (2.14)	3.67 (2.10)	**< 0.001**	0.63 (0.02, 1.24)	**0.045**
Every time naloxone administration to overdose	4.76 (2.09)	3.77 (2.11)	**< 0.001**	0.95 (0.41, 1.50)	**0.001**
Country at risk for HIV or HCV outbreak	4.73 (2.07)	4.15 (2.13)	**0.022**	0.26 (-0.28, 0.80)	0.348
Effectiveness of MOUD	4.73 (2.13)	3.79 (2.09)	**< 0.001**	1.11 (0.5, 1.71)	**< 0.001**
HCV screening offered	4.54 (2.17)	4.23 (2.13)	0.200	0.03 (-0.51, 0.56)	0.922
HIV screening offered	4.91 (2.17)	4.11 (2.09)	**0.002**	0.45 (-0.11, 1.01)	0.116

* Adjusted for demographic factors including age, sex, race, ethnicity, marital status, employment status, and income

## Discussion

This study found a relatively low overall harm reduction and recovery support score (HRRSS) in Oconee County, South Carolina, at a mean of 4.1, below half of the maximum HRRSS. This is consistent with the known low county-level scores on metrics of social capital and suggests that HRRSS could be indirectly measuring levels of social capital as it relates to OUD recovery potential, particularly as harm reduction and recovery support service organizations fall into the category of tangible social capital. Furthermore, the rates of positive response were smaller than 50% for 6 of the 9 HRRSS items. Nonetheless, HRRSS of younger and employed respondents are significantly greater than their older and unemployed or retired counterparts, although a further analysis revealed that the effect of the employment status was no longer significant after adjusting for age (data not shown). Respondents who agree that OUD is a disease and who agree with the effectiveness of MOUD also had greater HRRSS. In addition, respondents with less stigmatizing attitudes in general had significantly greater HRRSS.

A majority of survey respondents support placement of naloxone boxes in public places, condom distribution, and HIV and HCV screening for recovery support or harm reduction services. Although respondents are neutral for supporting the concept of SSP and SCSs, they appear to disagree somewhat strongly on locations and utilizations of SSP, financial support for SSP, and MOUD treatment for incarcerated population. The factors associated with support for SSP found in a sub-analysis of the 2019 OCORT data [34] were mostly replicated in the present analysis. While respondents perhaps understand the need for harm reduction resources such as SSPs or SCSs, they might also be somewhat resistant to building or placing physical resources to that end. Such resistance, quantified as lower HRRSS, is greater for those who disagree that OUD is a disease and that MOUD is effective, and is also greater for those who hold stigmatizing attitudes towards individuals with OUD. Thus, there is a clear and significant positive correlation among higher HRRSS, knowledge regarding OUD/SUD, and less stigmatizing attitudes toward individuals with OUD. These results reported in Table 4 were not significantly different when the employment status was adjusted for in the model even after further broken down into three categories of employed, seeking, and retired (data not shown).

In rural communities, the beneficial effects of SSPs and SCSs have not been well studied, although a qualitative analysis and systematic review [35] supported expansion of SSPs and SCSs into non-urban setting as evidence-based interventions to reduce overdose and transmission for infectious diseases. In other settings, however, the effectiveness of SSPs and SCSs has been well-documented with consistent evidence showing that availability of a SSP or SCS does not increase drug use or improper disposal of syringes but reduces overdose mortality and crime. For instance, SSPs reduced drug use and increased drug treatment enrollments in Seattle [36] and reduced infectious disease without increasing improper syringe disposal in high population areas such as California [37]. A study of SSPs in Baltimore indicated that after just 2 years of the program improper syringe disposal had been significantly reduced by > 46% [38]. Unsanctioned SCSs have succeeded in overdose prevention, infectious disease transmission reduction, and have even reduced crime in the United States [39, 40]. Similar findings have been reported in urban cities in Canada, Australia, Switzerland, and Spain, where studies focusing on supervised injection services reduced public drug injections and improper disposal of syringes without any increase in drug injecting, drug trafficking, crime or overdose mortality [41–43]. Naloxone programs have also substantially contributed to drug overdose reversals. From 2010 to 2014, local naloxone program sites increased from 188 to 644 (183% increase), distributions of naloxone from 53,032 to 152,283 (187% increase), overdose reversals from 10,171 to 26,453 (160% increase), and the participating states including Washington DC from 14 to 30 (94% increase) [44, 45].

Community harm reduction resources such as SSPs, SCSs, and naloxone distribution programs are tangible local social capital as it relates to mitigating the opioid overdose epidemic, while the attitudes toward these resources are intangible aspects of social capital in a community. A lack of support for these harm reduction resources in this study’s results, and the accompanying correlation between lack of support and stigmatizing attitudes suggests that the HRRSS is likely indirectly measuring social capital as it relates to attitudes and institutions that would improve OUD outcomes. Accordingly, increasing levels of OUD-related knowledge and decreasing stigmatizing attitudes in the community could be expected to increase support for harm reduction and recovery support resources, thus tangibly and intangibly increasing social capital levels. These findings highlight the importance of emphasizing education of the general population regarding the neurobiological (disease) model of OUD and other substance use disorders in general, along with communitywide educational efforts to reduce stigma towards individuals with OUD. If interventions can successfully target older and retired/unemployed persons, the improvement may be more marked. At the same time, to leverage an increase in social capital for mitigating the OUD epidemic, future research should strive to develop and evaluate comprehensive community-level multidimensional interventions targeting populations with high levels of stigma or with inadequate knowledge of OUD and its treatments to build resources for recovery services and harm reduction in our rural community.

A few limitations should be taken into consideration when the study findings are interpreted. First, the measure developed and reported in this study (HRRSS) has not been tested in any other population to date. Second, Oconee County may not be representative of US rural counties in terms of rurality, population composition or extent of OUD burden. This limits the generalizability of the findings to a broader rural or urban population. Third, our survey sampling strategy might have not resulted in a representative random sample of the Oconee County population as they might have been more motivated to respond by interest in the OUD epidemic. Collecting survey results in medical office waiting rooms may have contributed to the number of responses from women, as women are more likely to visit a doctor’s office. People with SUD or historically excluded groups are less likely to engage with formal institutions, including healthcare, and so also may have been underrepresented in this sample. Therefore, the implicated potential influence of response bias further limits generalizability of the findings. Furthermore, the dichotomized associations chosen for the analysis may have combined disparate groups within the community and affected results. All but two of the survey items read at an 8th grade level, but the other two read at a 9th grade level, so it is possible that readability of the survey affected results. Lastly, the “prefer not to answer” response was treated as missing, and its rate was approximately 11% for the potentially sensitive income item. Although the mechanism underlying any missing item is unknown, it was assumed to be missing completely at random.

## Conclusions

Building tangible or physical resources to improve recovery and harm reduction services and improving the intangible attitudes and knowledge that surround and affect the opioid overdose epidemic may mitigate the community destruction caused were the epidemic to spread unchecked in communities with low social capital. Although the need for these services appeared to be well recognized, community willingness to develop the social capital is inadequate as reflected in the low HRRSS reported in this study. Nonetheless, respondents with adequate knowledge of the disease model of OUD and the effectiveness of MOUD treatment – and those with lower levels of stigmatizing attitudes – have greater support for recovery and harm reduction services. Development and evaluation of comprehensive interventions to improve knowledge and reduce stigma and focusing more on older and unemployed or retired individuals could hold promise as a strategy to mitigate the opioid overdose epidemic in rural communities.

## Data Availability

The datasets used and analyzed during the current study are available from the corresponding author on reasonable request.

## Abbreviations

CI: Confidence Interval
DC: District of Columbia
HCV: Hepatitis C Virus
HIV: Human Immunodeficiency Virus
HRSS: Harm Reduction and Recovery Support Score
MOUD: Medications for Opioid Use Disorder
US: United States
OCORT: Oconee County Opioid Response Taskforce
OUD: Opioid Use Disorder
REDCap: Research Electronic Data Capture
SCS: Safe Consumption Site
SD: Standard Deviation
SSP: Syringe Service Program
